# 585. Safety of Pfizer-BioNtech COVID-19 Vaccine in Healthcare Workers, Singapore

**DOI:** 10.1093/ofid/ofab466.783

**Published:** 2021-12-04

**Authors:** Kai-Qian Kam, Chee Fu Yung, Chia Yin Chong, Jia hui Li, Karen Donceras Nadua, Natalie W Tan, Koh Cheng Thoon

**Affiliations:** KK Women's and Children's Hospital, Singapore, Not Applicable, Singapore

## Abstract

**Background:**

On 14 December 2020, the Pfizer-BioNTech coronavirus disease 2019 (COVID-19) vaccine was granted emergency use authorization in Singapore. Healthcare workers (HCW) were prioritized to receive the vaccine. We aim to investigate the side effects and risk factors for allergic reactions in our institution.

**Methods:**

All HCW vaccinations were recorded in an electronic centralized database. All reactions occurring within a 30-minute observation period post vaccination were recorded. Staff were required to report any vaccine-related medical consult including hospitalization occurring within 14 days after vaccination. Moderate/severe reactions were assessed by a medical team and determined if the reactions were probable allergic reactions with consultation with an Allergist. We extracted data from 8 Jan 2021 to 30 April 2021.

**Results:**

5030 and 159 HCW completed 2 doses and 1 dose of the vaccine respectively. There were 1056 HCWs (20.3%) with self-reported pre-existing allergy. There were 114 (1.1%) reactions occurring without the 30-minute observation period, and 64 (56.1%) were related to first dose of vaccine. The most common side effect experienced was aches or pain on any part of the body (n=46, 40.4%) followed by fatigue and/or giddiness (n=45, 39.5%), palpitations and/or shortness of breath (n=22, 19.3%), systemic rash and/or angioedema (n=12, 10.5%) and nausea and/or vomiting (n=12, 10.5%). A total of 23 HCWs complained of systemic rash and/or angioedema that occurred anytime post vaccination. Fifteen HCWs (0.29% of the cohort) were considered to have probable allergic reaction to the vaccine. None of the reactions were classified as anaphylaxis or severe reactions, but 4 HCWs required short hospitalization stay for observation. HCWs with pre-existing allergy had 2.6 times the risk of having probable vaccine-related allergic reaction than HCWs without pre-existing allergy (RR 2.6, 95% CI 0.9 to 7.3, p=0.068) but this was not statistically significant.

**Conclusion:**

No anaphylaxis or severe reactions were observed in our institution. Acute side effects in our cohort were in line with published trial reports. We noted a raised relative risk of 2.6 of pre-existing allergy with probable vaccine-related allergic reaction but this was not statistically significant.

**Disclosures:**

**All Authors**: No reported disclosures

